# Persistent asthma phenotype related with late-onset, high atopy, and low socioeconomic status in school-aged Korean children

**DOI:** 10.1186/s12890-017-0387-5

**Published:** 2017-02-23

**Authors:** Eun Lee, Si Hyeon Lee, Ji-Won Kwon, Young-Ho Kim, Jisun Yoon, Hyun-Ju Cho, Song-I Yang, Young-Ho Jung, Hyung Young Kim, Ju-Hee Seo, Hyo Bin Kim, So Yeon Lee, Ho-Jang Kwon, Soo-Jong Hong

**Affiliations:** 1 Department of Pediatrics, Chonnam National University Hospital, Gwangju, South Korea; 2 Asan Institute for Life Sciences, University of Ulsan College of Medicine, Seoul, South Korea; 3 Department of Pediatrics, Seoul National University Bundang Hospital, Seongnam, South Korea; 4 Department of Pediatrics, Gyeongsang National University Changwon Hospital, Changwon, South Korea; 5 Department of Pediatrics, Childhood Asthma and Atopy Center, Environmental Health Center, Asan Medical Center, University of Ulsan College of Medicine, 88 Olympic-ro 43 gil, Songpa-gu, Seoul, 05505 South Korea; 6 Department of Pediatrics, Hallym University Sacred Heart Hospital, Anyang, South Korea; 7 Department of Pediatrics, CHA University School of Medicine, Seongnam, South Korea; 8 Department of Pediatrics, Pusan National University Yangsan Hospital, Yangsan, South Korea; 9 Department of Pediatrics, Dankook University College of Medicine, Cheonan, South Korea; 10 Department of Pediatrics, Sanggye Paik Hospital, Inje University College of Medicine, Seoul, South Korea; 11 Department of Preventive Medicine, Dankook University College of Medicine, Cheonan, South Korea

**Keywords:** Asthma, Atopy, Children, Phenotype, Socioeconomic status

## Abstract

**Background:**

Treatment guidelines for asthma have been established based on asthma severity; there are limitations in the identification of underlying pathophysiology and prediction of prognosis in heterogeneous phenotypes of asthma. Although the complex interactions between environmental and genetic factors affect the development and progression of asthma, studies on asthma phenotypes considering environmental factors are limited. This study aimed to identify asthma phenotypes using latent class analysis including environmental factors in school-age children.

**Methods:**

We included 235 children (6–8 years) with parent-reported, physician-diagnosed asthma from the Children’s HEalth and Environmental Research (CHEER) study, which is a 4-year prospective follow-up study with 2-year intervals. At every survey, pulmonary function tests, methacholine challenge tests and blood tests with questionnaire were conducted.

**Results:**

Four asthma phenotypes were identified. Cluster 1 (22% of children) was characterized by high prevalence of atopy and mild symptoms; subjects in cluster 2 (17%) consisted of less atopy and normal lung function, but intermittent troublesome; cluster 3 (29%) experienced late-onset atopic troublesome asthma with decreased lung function in combination with low socioeconomic status; and cluster 4 was associated with early-onset and less-atopic infrequent asthma.

**Conclusions:**

Late-onset, high atopy, and low socioeconomic status are associated with troublesome persistent asthma phenotype in school-age children. Environmental factors might be implicated in the clinical heterogeneity of asthma. Asthma phenotypes considering diverse factors might be more helpful in the identification of asthma pathogenesis and its prevention.

**Electronic supplementary material:**

The online version of this article (doi:10.1186/s12890-017-0387-5) contains supplementary material, which is available to authorized users.

## Background

Asthma is characterized by airway hyperresponsiveness and reversible airway obstruction, which results from complex interactions between genetic and environmental factors [1]. Wheezing in early life is usually associated with viral respiratory tract infection; however, frequent recurrence in these groups causes a considerable healthcare burden [2]. Owing to the clinical and pathophysiological heterogeneity of asthma, there have been diverse attempts to phenotype asthma [3–7]. However, the actual clinical application of phenotyping asthma was based on symptom severity [8]. Classification of asthma is important to predict the prognosis and response to treatment and understand its underlying mechanisms; however, studies on asthma phenotypes using comprehensive factors are limited.

Epidemiologic studies have considered instability of asthma phenotypes over time [3, 4, 7] and identified associated risk factors for each asthma phenotype [9]. However, such studies considering environmental factors for the classification of asthma phenotypes are limited, although environmental factors play an important role as causative and provocative factors for asthma [10]. Additionally, few studies artificially classified asthma phenotypes according to onset age and/or persistence of wheezing, despite diverse factors affecting the development and progression of asthma [9, 11]. Environmental factors affect the development of airway during antenatal and/or postnatal period through epigenetic or non-epigenetic mechanisms [12, 13]. Although persistent troublesome wheeze is known to be associated with atopy, bronchial hyperresonsiveness (BHR), and decreased pulmonary function compared with controlled asthma [6, 14], these studies described phenotypes of preschool wheezing based on temporal patterns of symptoms combined with symptom control. Another study showed that sex is a risk factor for different asthma phenotypes [15]. However, these studies have limitations in identification of the underlying pathology according to phenotypes. No study has shown how environmental factors combined with genetic factors reflected in parental history of allergic diseases and demographic factors are associated with the heterogeneity of asthma, which would be helpful to investigate the origin of asthma.

The aim of this study was to identify asthma phenotypes based on environmental factors and clinical characteristics of asthma in school-age children using latent class analysis (LCA), which is an unsupervised statistical method for classification [16]. Additionally, we investigated the prognosis of each asthma phenotype and changes in biomarkers during the prospective follow-ups.

## Methods

### Study population

This study was a part of Children’s HEalth and Environmental Research (CHEER) study, which aimed to evaluate the effect of environmental factors on children’s health [16–18]. The study population was enrolled from January 2005 to December 2006, and they were followed-up for 4 years with 2-year intervals until December 2010 in randomly selected nationwide elementary schools in Korea [16, 17]. This cohort study consisted of parent-completed questionnaire, skin prick tests (SPTs), blood tests for total serum IgE levels and eosinophil percentages, pulmonary function tests, and methacholine challenge tests at every survey. The Korean version of the International Study of Asthma and Allergies in Childhood (ISAAC) questionnaire was used to assess the presence of allergic diseases [19–21]. Out of the total 2491 children at the time of enrollment in CHEER study, those (mean age ± standard deviation, 7.5 ± 0.9 years) with parent-reported, physician-diagnosed asthma in lifetime were included in the present study. The study protocols were reviewed and approved by the institutional review board (IRB) of Ulsan University College of Medicine (IRB no. 2006–0081). Written informed consent was obtained from the parents or guardians of the study participants Parents or guardians completed the questionnaire.

### Skin prick tests (SPTs)

The SPTs were performed for 13 common inhalant allergens and 4 food allergens [17]. Atopy was defined as at least one positive SPT response.

### Measurement of total serum IgE levels and blood eosinophil percentages

Total serum IgE levels were measured by using fluorescent enzyme immunoassay (AutoCAP system, Phadia AB, Uppsala, Sweden). The lower limit of total serum IgE detection was 2 kUA/L. Blood eosinophil percentages were evaluated using NE-8000 system (Sysmex, Kobe, Japan) from peripheral blood samples.

### Lung function test and BHR

Spirometry was performed according to the American Thoracic Society (ATS)/European Respiratory Society (ERS) guidelines by using a portable microspirometer (Microspiro HI-298, Chest Corporation, Tokyo, Japan) [17, 22, 23]. Details of BHR measurement are provided in elsewhere [24]. Subjects were considered to have BHR to methacholine when their methacholine PC_20_ was <8 mg/mL. The new development of BHR was defined as conversion from a negative BHR at enrollment to a positive BHR at the 4-year follow-up survey.

### Cluster analysis

Asthma phenotypes were identified by using LCA. LCA has been employed previously to identify phenotypes of asthma, atopic dermatitis (AD), and allergic rhinitis (AR) [16, 25, 26] because this statistical method can classify distinctly based on the observed heterogeneity in total population without bias. Several variables were used to define asthma phenotypes: demographic characteristics, namely, sex, body mass index (BMI), and socioeconomic status (SES) using monthly house hold income (http://stats.oecd.org) and maternal education level; and environmental factors, namely, prior exposure to environmental tobacco smoke (ETS), parental history of allergic diseases (AD, AR, and/or asthma), onset age of the first wheezing, number of absence days from school and nocturnal awakening days owing to asthma attacks, number of asthma attacks in the previous 12 months, number of days with treatment for asthma in the previous 12 months at the time of enrollment, and SPT, blood tests, and lung function tests at the time of enrollment. The FEV_1_ % predicted and FEV_1_/FVC were dichotomized as ≥80% and <80% and for FEF_25–75%_ % predicted as ≥65% and <65%. Based on the Bayesian information criteria, a model with four latent classes was selected as the best model for CHEER data [27].

### Statistical analysis

To evaluate the differences in characteristics and parameters among the four asthma phenotypes, we used the *t* test for independent comparisons between two groups, Fisher exact test for categorical variables, or Kruskal–Wallis test for continuous variables. Multiple testing was conducted using the Bonferroni correction when needed. *P* < 0.05 was considered significant. All statistical analyses were performed using SAS for Windows (version 9.2).

## Results

### Characteristics of study population

The characteristics of the study population are shown in Table 1. Mean age of the enrolled children was 7.5 years, and 63.4% of the study population were boys. The prevalence of parental history of allergic diseases, atopy, and physician-diagnosed AR and AD in lifetime was significantly higher in the included subjects compared to those excluded due to the absence of physician-diagnosed asthma history.

**Table 1:** Characteristics of study population

Variables	Participants in 4 clusters	Excluded in LCA	*P* value
Number	235/2491 (9.4%)	2256/2491 (90.6%)	
Male	149/235 (63.4%)	1148/2256 (50.9%)	<0.001
Age (years), mean ± SD	7.5 ± 0.9	7.7 ± 1.1	<0.001
BMI (kg/m^2^), mean ± SD	16.95 ± 2.4	16.87 ± 2.6	0.677
Parental allergic diseases, yes	97/227 (42.7%)	582/2114 (27.5%)	<0.001
Maternal educational levels, ≤ High school	133/222 (59.9%)	1261/1967 (64.1%)	0.218
Parental monthly income, < 3000 USD	152/230 (66.1%)	1501/2132 (70.4%)	0.175
ETS exposure, yes	106/227 (46.7%)	910/2004 (45.4%)	0.712
Atopy on skin prick tests	104/234 (44.4%)	583/2254 (25.9%)	<0.001
Total serum IgE (IU/ml), mean [range]	454 [3.80–4630]	250.80 [1.40–6934]	<0.001
Blood eosinophils (%), mean [range]	4.67 [0.50–39.10]	3.57 [0–22.60]	<0.001
AR diagnosis, ever	107/234 (45.7%)	462/2081 (22.2%)	<0.001
AD diagnosis, ever	99/233 (42.5%)	642/2075 (30.9%)	<0.001
FEV_1_ % predicted	102.4 ± 13.77	106.1 ± 12.96	<0.001
FEV_1_/FVC	88.86 ± 6.06	90.86 ± 5.54	<0.001
FEF_25–75%_ % predicted	91.92 ± 21.88	99.91 ± 22.64	<0.001
BHR (PC_20_ < 8 mg/ml)	80/221 (36.2%)	408/2204 (18.5%)	<0.001

Data are presented as number (%) or mean ± standard deviation

*Definition of abbreviations: AD* atopic dermatitis, *AR* allergic rhinitis, *BHR* methacholine PC20 < 8mg/ml, *BMI*, body mass index, *ETS* environmental tobacco smoke, *FEF*_*25–75%*_ Forced expiratory flow at 25–75% of forced vital capacity, *FEV*_*1*_ Forced expiratory volume in 1 s, *FVC* Forced vital capacity, *LCA﻿* latent class analsis, *SD* standard deviation, *USD* US dollars

### Cluster 1 (atopic mild asthma)

Fifty-one children (21.7%) were grouped into cluster 1, which was characterized by atopic mild asthma (Table 2). Atopy was observed in 46 children (90.2% of the children in this cluster). Onset age of the first wheezing in this cluster ranged widely from ≤ 1 year to > 6 years of age. None of the children in this cluster complained of absence days from school and nocturnal awakening days due to asthma attacks in the previous 12 months at the time of enrollment. Forty-two children (82.4%) had no asthma attacks in the previous 12 months at the time of enrollment. However, BHR was observed for 44.9% of the children (*n* = 22/49) and > 100 IU/mL of total serum IgE levels for 98.0% (*n* = 50/51).

**Table 2:** Results of latent class analysis

Cluster	Cluster 1	Cluster 2	Cluster 3	Cluster 4
Number, n (%)	51 (21.7%)	40 (17.0%)	29 (12.3%)	115 (49.1%)
Sex, Male	28/51 (54.9%)	30/40 (75.0%)	14/29 (48.3%)	77/115 (67.0%)
Body mass index				
Normal	43/51 (84.3%)	31/39 (79.5%)	29/29 (100.0%)	88/111 (79.3%)
Overweight	8/51 (15.7%)	1/39 (2.6%)	0/29 (0.0%)	16/111 (14.4%)
Obesity	0/51 (0.0%)	7/39 (17.9%)	0/29 (0.0%)	7/111 (6.3%)
Family history of allergic diseases, yes	28/50 (56.0%)	14/38 (36.8%)	16/26 (61.5%)	39/113 (34.5%)
Monthly income, < 3000 USD	32/49 (65.3%)	21/40 (52.5%)	26/28 (92.9%)	73/113 (64.6%)
Maternal educational level				
Less than high school	30/46 (65.2%)	15/36 (41.7%)	21/29 (72.4%)	67/111 (60.4%)
Exposure to ETS, yes	12/50 (24.0%)	20/38 (52.6%)	17/28 (60.7%)	57/111 (51.4%)
Atopy, yes	46/51 (90.2%)	18/40 (45.0%)	27/29 (93.1%)	13/114 (11.4%)
Onset age of first wheezing				
< 1 year	10/44 (22.7%)	5/35 (14.3%)	0/28 (0.0%)	18/103 (17.5%)
1–3 years	9/44 (20.5%)	9/35 (25.7%)	6/28 (21.4%)	46/103 (44.7%)
3–6 years	11/44 (25.0%)	14/35 (40.0%)	17/28 (60.7%)	29/103 (28.2%)
≥ 6 years	14/44 (31.8%)	7/35 (20.0%)	5/28 (17.9%)	10/103 (9.7%)
Number of absence days from school due to wheezing^a^				
0	51/51 (100.0%)	24/40 (60.0%)	18/29 (62.1%)	115/115 (100.0%)
1–3 days	0/51 (0.0%)	15/40 (37.5%)	5/29 (17.2%)	0/115 (0.0%)
4–6 days	0/51 (0.0%)	0/40 (0.0%)	4/29 (13.8%)	0/115 (0.0%)
≥ 7 days	0/51 (0.0%)	1/40 (2.5%)	2/29 (6.9%)	0/115 (0.0%)
Number of nocturnal awakening days due to wheezing^a^				
0	51/51 (100.0%)	13/40 (32.5%)	9/29 (31.0%)	115/115 (100.0%)
< 1 /week	0/51 (0.0%)	24/40 (60.0%)	15/29 (51.7%)	0/115 (0.0%)
≥ 1 /week	0/51 (0.0%)	3/40 (7.5%)	5/29 (17.2%)	0/115 (0.0%)
Number of asthma attack^a^				
0	42/51 (82.4%)	0/40 (0.0%)	0/29 (0.0%)	108/115 (93.9%)
1–3	9/51 (17.6%)	26/40 (65.0%)	11/29 (37.9%)	7/115 (6.1%)
4–12	0/51 (0.0%)	8/40 (20.0%)	7/29 (24.1%)	0/115 (0.0%)
≥ 13	0/51 (0.0%)	6/40 (15.0%)	11/29 (37.9%)	0/115 (0.0%)
Asthma treatment^a^, yes	19/51 (37.3%)	32/40 (80.0%)	27/29 (93.1%)	21/115 (18.3%)
Total serum IgE ≥100 IU/mL	50/51 (98.0%)	24/40 (60.0%)	28/28 (100.0%)	30/111 (27.0%)
Blood eosinophil ≥4%	28/51 (54.9%)	24/40 (60.0%)	23/29 (79.3%)	29/112 (25.9%)
BHR <8 mg/ml	22/49 (44.9%)	12/38 (31.6%)	21/26 (80.8%)	25/108 (23.1%)
FEV_1_ < 80% predicted	7/47 (14.9%)	0/39 (0.0%)	4/29 (13.8%)	1/113 (0.9%)
FEV_1_/FVC <80% predicted	1/49 (2.0%)	0/40 (0.0%)	11/29 (37.9%)	8/114 (7.0%)
FEF_25–75%_ <65% predicted	5/29 (17.2%)	0/30 (0.0%)	8/19 (42.1%)	4/90 (4.4%)

^a^in the previous 12 months

*Definition of abbreviations: BHR* methacholine PC20 < 8mg/ml, *ETS* environmental tobacco smoke, *FEF*_*25–75%*_ Forced expiratory flow at 25–75% of forced vital capacity, *FEV*_*1*_ Forced expiratory volume in 1 s, *FVC* Forced vital capacity, *USD* US dollars

### Cluster 2 (less atopic, troublesome asthma)

Forty children (17.0% of the enrolled children) were assigned to cluster 2, characterized by normal lung function with moderately troublesome asthma with high SES, reflected by monthly household income and maternal educational levels. Obesity was observed in 17.9% of the children (*n* = 7/39) in this cluster. This cluster had the lowest prevalence of low monthly income (*n* = 21/40, 52.5%) and lower levels of maternal educational levels (*n* = 15/36, 41.7%). All the children in this cluster experienced wheezing more than once in the previous 12 months at the time of enrollment. Sixteen children (40%) of this cluster experienced absence days from school due to asthma attacks in the previous 12 months, and 27 children (67.5%) experienced nocturnal awakening days due to asthma attacks in the previous 12 months at the time of enrollment. However, all the children had normal lung function. Out of 38, 12 children (31.6%) in this cluster showed positive BHR.

### Cluster 3 (atopic, persistently troublesome asthma)

Twenty–nine children (12.3%) were grouped in this cluster. Children in this cluster experienced their first wheezing after 1 year of age. All children had normal BMI. Further, the highest prevalence of low SES was observed. The quality of life due to asthma attacks was poorest in this cluster; all the children experienced wheezing in the previous 12 months, and 27 children (93.1%) were treated for asthma attacks in the previous 12 months at the time of enrollment; 11 children (37.9%) were reported to have absence days from school due to asthma attacks and 20 children (69.0%) experienced nocturnal awakening owing to asthma attacks in the previous 12 months at the time of enrollment. The total serum IgE levels were >100 IU/mL for all the children, and blood eosinophil percentage was >4% in 23 children (79.3%). Out of 26 children, 21 (80.8%) showed positive BHR, and four of the total children showed <80% of FEV_1_ % predicted.

### Cluster 4 (less-atopic mild asthma)

One hundred fifteen children (48.9%) were included in this cluster, which was the largest. The prevalence of atopy and parental history of allergic diseases including AD, AR, and asthma was the lowest (*n* = 39/113, 34.5%; *n* = 13/114, 11.4%, respectively). Seven children (6.1%) experienced asthma attacks, although 21 children (18.3%) were treated due to asthma exacerbation during the previous 12 months at the time of enrollment. However, none of the children in this cluster were reported to have any absence days from school or nocturnal awakening days due to asthma attacks in the previous 12 months at the time of enrollment. The prevalence of ≥100 IU/ml of total serum IgE levels (*n* = 30/111; 27.0%) and ≥4% of blood eosinophil percentages (*n* = 29/112; 25.9%) was the lowest in this cluster. Additionally, the prevalence of BHR was the lowest (*n* = 25/108; 23.1%), and most of the children in this cluster showed normal lung function.

### Comorbidities

The prevalence of parent-reported, physician-diagnosed AR in lifetime was the highest in cluster 4 (60.0%), followed by clusters 2 (57.5%) and 1 (56.0%) (Table 3). The prevalence of parent-reported, physician-diagnosed AD in lifetime was the highest in cluster 3 (55.2%), followed by clusters 2 (50.0%) and 4 (39.5%). The prevalence of parent-reported, physician-diagnosed food allergy in lifetime was the highest in cluster 2 (15.4%), followed by cluster 4 (12.4%), although no significant differences were observed among the clusters.

**Table 3:** Comorbidities of allergic diseases at the time of enrollment according to asthma clusters

	Cluster 1	Cluster 2	Cluster 3	Cluster 4	*P* value
Allergic rhinitis	28/50 (56.0)	23/40 (57.5)	00/29 (34.5)	46/115 (60.0)	0.061
Atopic dermatitis	18/50 (36.0)	20/40 (50.0)	16/29 (55.2)	45/114 (39.5)	<0.001
Food allergy	2/49 (4.1)	6/39 (15.4)	2/28 (7.1)	14/113 (12.4)	0.269

### Biomarkers

Mean values of the total serum IgE and blood eosinophil percentages showed a decreasing pattern as the children grew in each cluster (Table 4). The total serum IgE levels and blood eosinophil percentages were the highest in cluster 3, followed by clusters 2, 4, and 1. This decreasing pattern was observed even at the final survey.

**Table 4:** Total serum IgE levels and blood eosinophil percentages at the time of enrollment and follow-up

Variables		At the time of enrollment					Follow-up				
		Cluster 1	Cluster 2	Cluster 3	Cluster 4	*P* value	Cluster 1	Cluster 2	Cluster 3	Cluster 4	*P* value
Total serum IgE levels (IU/mL)	mean (N)	746.8 (51)	306.1 (40)	1238.0 (28)	175.0 (111)	<0.001	378.9 (35)	148.7 (22)	447.7 (17)	77.2 (68)	<0.001
	95% CI	519.3–974.3	125.4–486.9	738.0–1738.0	87.7–262.6		296.8–460.9	86.0–211.4	268.3–627.1	45.0–109.5	
Blood eosinophil, %	mean (N)	5.7 (51)	5.3 (40)	6.2 (29)	3.6 (112)	<0.001	4.4 (35)	3.8 (22)	5.4 (17)	3.1 (69)	0.002
	95% CI	4.6–6.8	4.1–6.5	4.9–7.6	2.8–4.3		3.5–5.3	2.5–5.1	3.8–7.0	2.7–3.6	

*Definition of abbreviations: CI* confidence intervals, *N* number

**Fig. 1:**
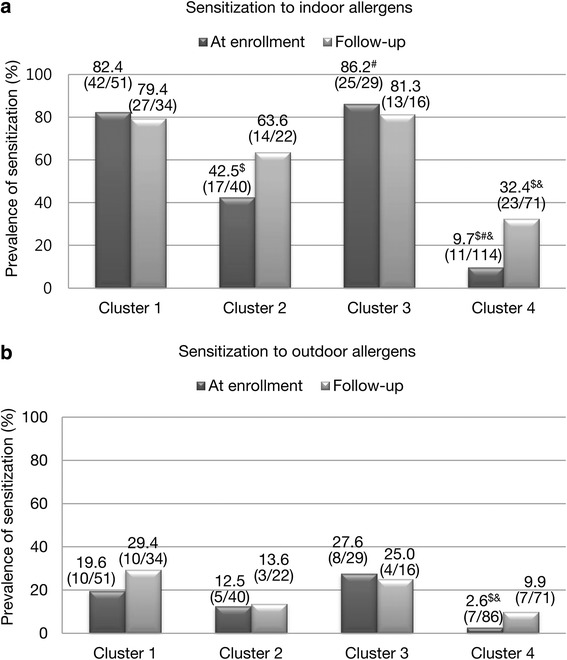
Sensitization according to asthma phenotypes. **a** Sensitizations to indoor allergens both at the time of enrollment and follow-up. **b** Sensitizations to outdoor allergens both at the time of enrollment and follow-up. ^$^ *P* value < 0.005, compared with cluster 1. ^#^ *P* value < 0.005, compared with cluster 2. ^&^ *P* value < 0.005, compared with cluster 3

The prevalence of sensitization to indoor allergens was the highest in cluster 3 (*n* = 25/29; 86.2%), followed by clusters 1 (*n* = 42/51; 82.4%), 2 (*n* = 17/40; 42.5%), and 4 (*n* = 11/114; 9.7%) at the time of enrollment (Fig. 1). The statistical differences in the prevalence of sensitization were observed between clusters 2 and 3 and between clusters 1 and 4. The same pattern was observed at the final survey with no statistical differences between clusters 2 and 3. Cluster 3 showed the highest prevalence of sensitization to outdoor allergens (*n* = 8/29; 27.6%) at the time of enrollment without statistical differences.

### Lung function

**Fig. 2:**
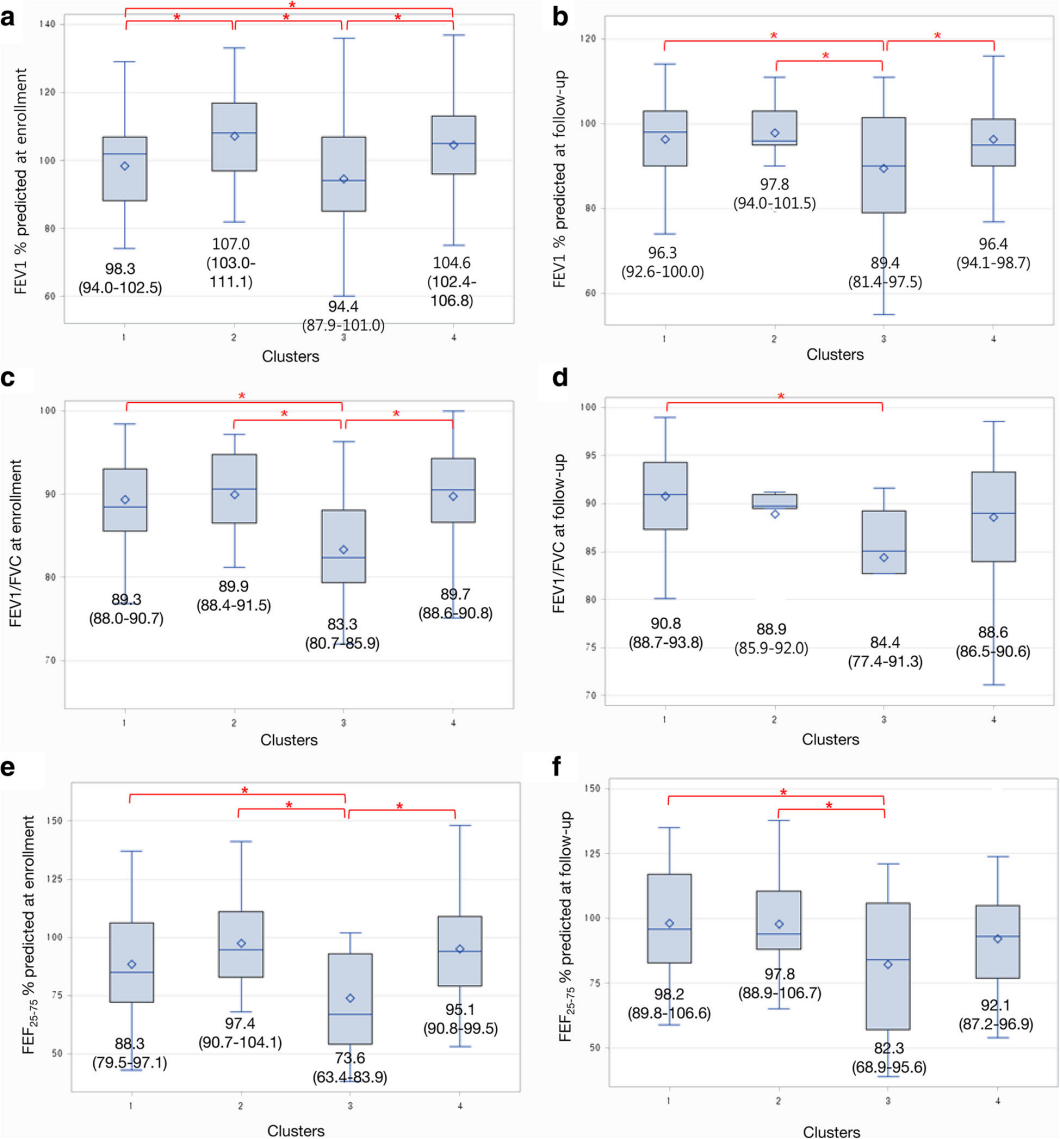
Changes in pulmonary function according to asthma clusters. **a** FEV_1_ % predicted at the time of enrollment. **b** FEV_1_ % predicted at the time of follow-up. **c** FEV_1_/FVC % predicted at the time of enrollment. **d** FEV_1_/FVC % predicted at the time of follow-ups. **e** FEF_25–75%_ % predicted at the time of enrollment. **f** FEF_25–75%_ % predicted at the time of follow-ups. ^*^ *P* value < 0.05

At the time of enrollment, mean values of FEV_1_ % predicted in cluster 3 (mean, 94.4; 95% confidence intervals [CI], 87.9–101.0) were significantly lower than that in cluster 1 (mean, 98.3; 95% CI, 94.0–102.5), compared with clusters 2 (mean, 107.0; 95% CI, 103.0–111.1) and 4 (mean, 104.6; 95% CI, 102.4–106.8) (Fig. 2). This pattern in FEV_1_ % predicted was also observed at the final survey. Mean levels of FEV_1_/FVC % predicted in cluster 3 (mean, 83.3; 95% CI, 80.7–85.9) were significantly lower compared with those in cluster 2 (mean, 89.9; 95% CI, 88.4–91.5) at the time of enrollment. However, no significant differences in FEV_1_/FVC % predicted between clusters 2 (mean, 88.9; 95% CI, 85.9–92.0) and 3 (mean, 84.4; 95% CI, 77.4–91.3) were observed, although FEV_1_/FVC % predicted was the lowest in cluster 3 at the final survey.

The levels of FEF_25–75%_ % predicted were lowest in cluster 3. The levels of FEF_25–75%_ % predicted were significantly lower in cluster 3 compared with those in cluster 2 both at the time of enrollment and at the final survey. However, no significant differences in the levels of FEF_25–75%_ % predicted were observed between clusters 1 and 4 both at the time of enrollment and at the final survey.

### Prognosis

The prevalence of new-onset BHR during the prospective follow-ups was the highest in cluster 3 (*n* = 3/5; 60.0%), followed by clusters 1 (*n* = 8/21; 38.1%) and 4 (*n* = 5/64; 7.8%) (Table 5). The persistence rate of BHR was the highest in cluster 1 (*n* = 12/20; 60.0%), followed by cluster 3 (*n* = 7/15; 46.7%). The remission rate of BHR was the highest in cluster 4 (*n* = 20/22; 90.9%), followed by cluster 2 (*n* = 6/7; 85.7%). The prevalence of asthma attacks during the previous 12 months at follow-ups was the highest in cluster 3 (*n* = 13/24; 54.2%), followed by cluster 2 (*n* = 10/26; 38.5%).

**Table 5:** Prognosis of asthma phenotypes according to asthma clusters

Variables	Cluster 1	Cluster 2	Cluster 3	Cluster 4	*P* value
New-onset BHR, n (%)	8/21 (38.1)	1/17 (5.9)	3/5 (60.0)	5/64 (7.8)^*^	<0.001
Persistence of BHR, n (%)	12/20 (60.0)	1/7 (14.3)	7/15 (46.7)	2/22 (9.1)^*^	<0.002
Remission of BHR, n (%)	8/20 (40.0)	6/7 (85.7)	8/15 (53.3)	20/22 (90.9)^*^	0.002
Wheezing in the previous 12 months at follow-up, n (%)	4/23 (17.4)	10/26 (38.5)	13/24 (54.2)	3/25 (12.0)†	0.005
New-onset AR, n (%)	9/18 (50.0)	6/9 (66.7)	7/15 (46.7)	15/60 (25.0)	0.026
New sensitization on SPT	13/42 (31.0)	4/26 (15.4)	6/23 (26.1)	27/91 (29.7)	0.493

*Definition of abbreviations: AR* allergic rhinitis, *BHR* methacholine PC20 < 8 mg/ml, *SPT* skin prick test

^*^ *P* value < 0.005, compared with cluster 1

^†^ *P* value < 0.005, compared with cluster 3

## Discussion

In the present prospective general-population based study, we identified four distinct asthma clusters in school-age children (Additional file 1: Figure S1). More frequent asthma attacks in the previous 12 months at the time of enrollment were observed in two asthma phenotypes (clusters 2 and 3) among the four clusters. However, the two clusters differed in SES; cluster 3 included children with lower SES, whereas prevalence of high SES was higher in cluster 2. Additionally, cluster 3 had a higher prevalence of atopy combined with an increased prevalence of BHR and decreased lung function. The other two clusters (clusters 1 and 4) included less frequent asthma attacks; however, they differed in atopy prevalence. The higher prevalence of atopy and ≥100 IU/mL of total serum IgE was observed in cluster 1. However, 17.6% of the children in cluster 1 had experienced asthma attacks in the previous 12 months at the time of enrollment; positive BHR was observed in about 45%. These findings suggest that asthma phenotypes in children can be determined based on atopy and SES combined with symptom severity. The comprehensive consideration of diverse factors might contribute to the identification of the origin of asthma. To our knowledge, this study is the first to identify asthma phenotypes using severity, atopy burden, and SES in school-age children.

Guidelines for asthma treatment were suggested based on asthma severity [8]. However, this classification lacks in the reflection of heterogeneity in asthma phenotypes, especially in children with unpredictable prognosis as they grew. To identify the phenotypic heterogeneity over time in children with asthma, strenuous efforts have been made via the epidemiologic studies [6, 9, 23, 28, 29]. The previous studies classified wheezing phenotype according to onset age of wheezing and/or persistence of asthma symptoms, especially in preschool children [29, 30]. Another study has identified wheezing phenotypes based on symptom patterns such as episodic viral or multiple-trigger wheeze in preschool children [31]. Although these phenotypes have contributed to the prediction of prognosis in preschool children with wheezing, the previous studies could not identify the underlying complex pathologies in each phenotype; because associated factors were partially considered for the heterogeneity of asthma.

We included BMI, parental history of allergic diseases, lung function, atopy, asthma symptoms, and SES for LCA to better understand the complex interactions between environmental and genetic factors according to asthma phenotypes and to identify the underlying mechanism. SES, one of the differentiating points in two troublesome asthma phenotypes in the present study, has been identified to have an inverse association with asthma regardless of age [32]. In another study, low-income levels were associated with the development and persistency of asthma [33–35]. Although the mechanisms underlying the association between SES and asthma have not been clearly identified, this factor might affect the pathology of asthma via complex interactions with other factors in some phenotypes of asthma, as identified in this study. This might be partially explained by inconsistent associations determined between SES and asthma [36]. The association between SES and asthma can be partially explained by exposure degree to allergens, gene by environmental interactions, nutrition, and psychosocial factors [37–39]. These factors are considered to affect the immune development through balance between Th1 and Th2 immune response [40, 41], which might be linked to alteration in microbiota in humans [42–44]. Alterations of colonization in gut or airway might affect the development and regulation of immune system with shaping of airway structures and gene expression through gene-environment interactions; therefore, they might contribute to the development and severity of asthma [45–48]. Also, low SES might affect the control of asthma in the aspect of adherence or compliance to asthma treatment. However, higher levels of total serum IgE as well as eosinophil (%) both at the time of enrollment and follow-up might imply the different pathophysiologies underlying more severe asthma, as shown in cluster 3.

The previous studies have identified that atopy, late-onset wheeze, and/or BHR are associated with persistent or severe asthma in children [6, 11, 30]. However, in another study, multiple trigger wheezing is associated with decreased pulmonary function regardless atopy or current wheezing [5]. These contrasting findings suggest that atopy alone might not explain the severity and heterogeneity in asthma; however, it can differently affect the prognosis of asthma depending on phenotypes. In the present study, cluster 1 was characterized by high prevalence of atopy similar to cluster 3, in which atopy was highly prevalent with troublesome and persistent asthma; however, children in cluster 1 had mild symptoms of asthma. Based on the previous and present studies [5, 14], certain phenotypes of asthma are influenced by complex combinations that affect interactions between innate and adaptive immunity [49, 50]; therefore, this might contribute to the understanding of the mechanisms and the development of individualized treatment according to asthma phenotypes.

The total serum IgE levels and blood eosinophil percentages had similar patterns with degrees of atopy and BHR, rather than asthma severity, similar to the previous study [51]. The assessments of these biomarkers help to identify asthma phenotypes with partial explanation for the underlying pathology in these phenotypes. However, high atopy is not equal to persistence or severity of asthma, as reflected in cluster 1 in the present and previous studies [4, 5]; it would be more helpful to consider the more complex factors in the prediction of prognosis in each phenotype of asthma.

In contrast to the previous studies on wheezing phenotypes in preschool children [3, 6, 11, 14], a wide overlap in onset-age of the first wheezing was observed across four asthma phenotypes in the present study. Overlapping features are commonly encountered in the results of LCA and this might practically reflect the real phenomenon in the classification of asthma. In the previous studies, late-onset asthma was classified as an independent phenotype regardless of persistency of asthma [6, 14]. This might suggest that onset-age of the first wheezing does not contribute to the differences in underlying mechanisms according to asthma phenotypes.

Discrepancy in frequency of asthma symptoms and treatment in the previous 12 months at the time of enrollment across asthma phenotypes might be attributable to the early application of asthma treatment in the presence of asthma like symptoms such as cough. The prevalence of this discrepancy was highest in cluster 4, which was characterized by less atopic mild asthma phenotype. This classification might help to predict the treatment behavior of asthma in each phenotype, and thereby, facilitate the targeted therapy and patient education based on asthma phenotypes. This might be useful to reduce the socioeconomic burden owing to patient morbidity and mortality in asthma [52].

There are some limitations in this study. Retrospective recall of childhood events has limitations in accurate assessment; however, this study included a prospective follow-up with 2-year intervals for 4 years in school-age children selected from a nationwide general population. Physician-diagnosed asthma was also based on parent reports. However, the prevalence of asthma diagnosis in the present study was 9.4% (*n* = 235/2491), which is compatible with the worldwide prevalence of asthma (6–9%) [53]. Follow-ups were relatively short. The assessment on treatment pattern and responses to treatment was lacking. However, disease burden was assessed using questions on the frequency of nocturnal awakening days and absence days from school owing to asthma attacks; this information can indirectly reflect the diseases course. Moreover, we enrolled nationwide school-age children with asthma; therefore, the results of this study can be generalized. Although this study included several environmental factors for cluster analysis, further asthma phenotypes including index representatives of air pollution are needed.

## Conclusion

We identified four asthma phenotypes in school-age children based on severity of asthma, SES, and atopy. Persistently troublesome asthma was divided into two phenotypes based on SES with atopy. Mild, intermittent asthma was divided into two phenotypes according to the degree of atopy. These findings suggest the importance of factors that affect SES in the classification of asthma phenotypes to identify the mechanisms underlying the heterogeneous asthma phenotypes. Consideration of diverse factors including environmental factors might be more helpful to identify the underlying pathogenesis according to asthma phenotype, prevent progression of asthma, and elucidate targeted therapy.

## Additional file

Additional file 1: Figure S1. Tree analysis using latent class analysis in four asthma phenotypes. *Definition of abbreviations: BHR* methacholine PC20 < 8 mg/ml. (TIF 736 kb)

## Abbreviations

AD: Atopic dermatitis
AR: Allergic rhinitis
BHR: Bronchial hyperresponsiveness
CHEER: Children’s HEalth and Environmental Research
ETS: Environmental tobacco smoke
FA: Food allergy
FEF_25–75%_: Forced expiratory flow at 25–75% of forced vital capacity
FEV_1_: Forced expiratory volume in 1 second
FVC: Forced vital capacity
PC_20_: Cumulative methacholine dose that caused a 20% reduction in FEV_1_
SES: Socioeconomic status
SPT: Skin prick test.
