# Supplementary figures and images for: Persistent asthma phenotype related with late-onset, high atopy, and low socioeconomic status in school-aged Korean children
Source: BMC Pulm Med. 2017 Feb 23;17:45. doi: 10.1186/s12890-017-0387-5 (PMC5324247; doi:10.1186/s12890-017-0387-5)

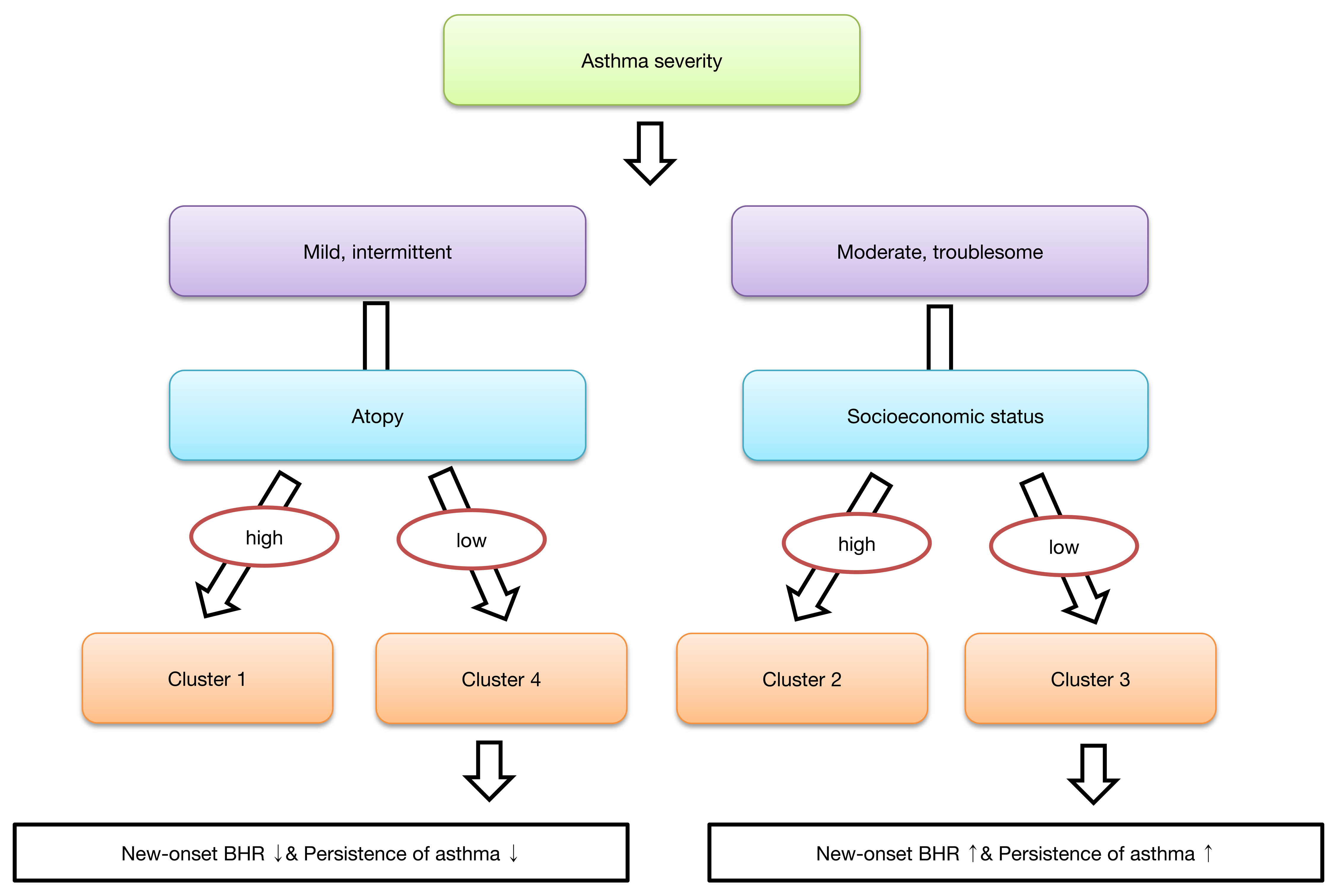

Supplement: Additional file 1: Figure S1. — Tree analysis using latent class analysis in four asthma phenotypes. Definition of abbreviations: BHR methacholine PC20 < 8 mg/ml. (TIF 736 kb) [file 12890_2017_387_MOESM1_ESM.tif]
